# Artificial Intelligence- and Machine Learning-Assisted Structure-Based Virtual Screening of Compounds That Target 15PGDH

**DOI:** 10.3390/pharmaceutics18080957

**Published:** 2026-08-03

**Authors:** Syed Sayeed Ahmad, Inho Choi

**Affiliations:** 1 Department of Medical Biotechnology, Yeungnam University, Gyeongsan 38541, Republic of Korea; sayeedahmad4@gmail.com; 2 Research Institute of Cell Culture, Yeungnam University, Gyeongsan 38541, Republic of Korea

**Keywords:** machine learning, screening, molecular dynamics simulation, 15PGDH, skeletal muscle

## Abstract

**Background**: Skeletal muscle (SM) plays a critical role in movement, metabolism, and organ protection, with its maintenance and regeneration relying on muscle satellite (stem) cells (MSCs). Prostaglandin E2 (PGE2) regulates MSCs, but PGE2 levels decline with aging due to increased catabolism by 15-hydroxyprostaglandin dehydrogenase (15PGDH), a negative regulator of muscle repair. **Methods**: This study aimed to employ artificial intelligence and machine learning (ML)-assisted, structure-based screening approaches to identify novel 15PGDH inhibitors. Supervised models (support vector machine, random forest, and XGBoost were trained on curated bioactivity data (IC_50_ values) from the ChEMBL database and used to virtually screen the Maybridge compound library (~51,000 compounds). **Results**: The area under the curve (AUC) values of the developed models SVM, RF, and XGBoost were 0.96, 0.99, and 1.00, respectively. Promising inhibitors were further validated using structure-based virtual screening (docking), molecular dynamics simulations (200 ns), and MM-PBSA/GBSA analyses. The top five inhibitors (PD00616, HTS11491, HTS02629, AW00889, and HTS11190) were identified as active (ML analysis) and potential 15PGDH inhibitors based on their subsequent binding affinities, involvement of catalytic residues (Ser138, Tyr151, and Lys155), and complex stability. Additionally, these inhibitors were found to follow the drug-likeness criteria. **Conclusions**: These findings offer valuable insights for the development of novel therapeutics targeting 15PGDH to combat muscle degeneration and related pathologies, including aging and sarcopenia.

## 1. Introduction

Skeletal muscle (SM) is a vital tissue responsible for maintaining posture and movement, regulating metabolism, and protecting organs. SM is largely involved in muscle contraction, thermogenesis, and myokine secretion [1,2]. SM is primarily generated by the paraxial mesoderm and undergoes tightly coordinated development through myogenesis, which involves the activation and proliferation of muscle satellite (stem) cells (MSCs), and the fusion of differentiating myoblasts into mature myofibers under the control of muscle regulatory factors [3] and growth factors [4].

Muscle mass and function maintenance depend on the balance between protein synthesis and degradation. Muscle wasting occurs when this balance is disrupted, leading to increased myofibrillar protein breakdown. Under normal conditions, SM remains relatively stable; however, in response to exercise, injury, disease, or aging, muscle tissue may undergo atrophy, hypertrophy, or even myofiber death [1,5]. Muscle hypertrophy occurs when protein synthesis exceeds protein degradation, a process regulated positively by the IGF-1–Akt–mTOR pathway and negatively by the myostatin–Smad2/3 pathway. Conversely, muscle atrophy results when protein degradation exceeds protein synthesis, often triggered by conditions such as starvation, denervation, cancer cachexia [6], heart failure, and aging. Two key protein degradation pathways are activated during muscle atrophy: the proteasomal and autophagic–lysosomal pathways [5]. Additionally, SM contains a diverse population of MSCs that are essential for muscle maintenance and regeneration and are positively regulated by prostaglandin E2 (PGE2). Meanwhile, PGE2 levels decline in aged SM due to increased prostaglandin catabolism mediated by 15-hydroxyprostaglandin dehydrogenase (15PGDH), a known negative regulator of muscle repair and regeneration that is ubiquitously expressed in mammalian cells. Notably, 15PGDH catalyzes the degradation of prostaglandins, particularly PGE2, by rapidly oxidizing 15-hydroxy-PGs into biologically less active 15-keto-PGs [7], which are unable to bind to prostaglandin receptors. PGE2 exerts protective effects on muscle by promoting muscle protein synthesis and reducing inflammation [8]. Growing evidence indicates that 15PGDH plays critical roles in multiple physiological and pathological processes, making 15PGDH a promising pharmacological target [9] for preventing organ damage, enhancing tissue regeneration, improving bone marrow graft recovery, and counteracting aging-associated tissue dysfunction [10]. Therefore, 15PGDH inhibition is predicted to elevate PGE2 levels and promote healing and tissue regeneration in vivo [11]. A study of a 15PGDH knockout mice found that the animals were healthy and exhibited a normal lifespan [12]. These studies suggest that optimized 15PGDH inhibitors could safely elevate PGE2 levels in vivo and accelerate the recovery of multiple tissues. Overall, 15-hydroxy-PGs, such as PGE2, support MSC activation, proliferation, and muscle repair in normal muscle. In contrast, increased 15PGDH activity in aging muscle reduces PGE2 levels, thereby impairing MSC function and hindering muscle regeneration, contributing to age-related muscle atrophy. Thus, targeting 15PGDH or modulating PGE2 levels could offer therapeutic strategies to mitigate muscle loss during aging. Hence, this study aimed to integrate artificial intelligence (AI) and machine learning (ML) approaches to identify novel 15PGDH inhibitors. The Maybridge compound library was selected as a chemically diverse dataset for the virtual screening of potential 15PGDH inhibitors. Supervised ML algorithms, including support vector machines (SVMs), random forests (RFs), and extreme gradient boosting (XGBoost), were employed to develop predictive classification models. Experimentally reported 15PGDH bioactivity data were retrieved from the ChEMBL database and curated to generate high-quality training and test datasets. Molecular descriptors and fingerprints were calculated and used as input features for model development. Model performance was rigorously evaluated and optimized on the test dataset using appropriate validation metrics. The best-performing and optimized models were subsequently applied to virtually screen the Maybridge library, enabling the identification of computational hits against 15PGDH [13] via structure-based virtual screening, molecular dynamics (MD) simulations, and MM-PBSA/GBSA analyses.

## 2. Materials and Methods

### 2.1. Machine Learning Datasets

**Figure 1:**
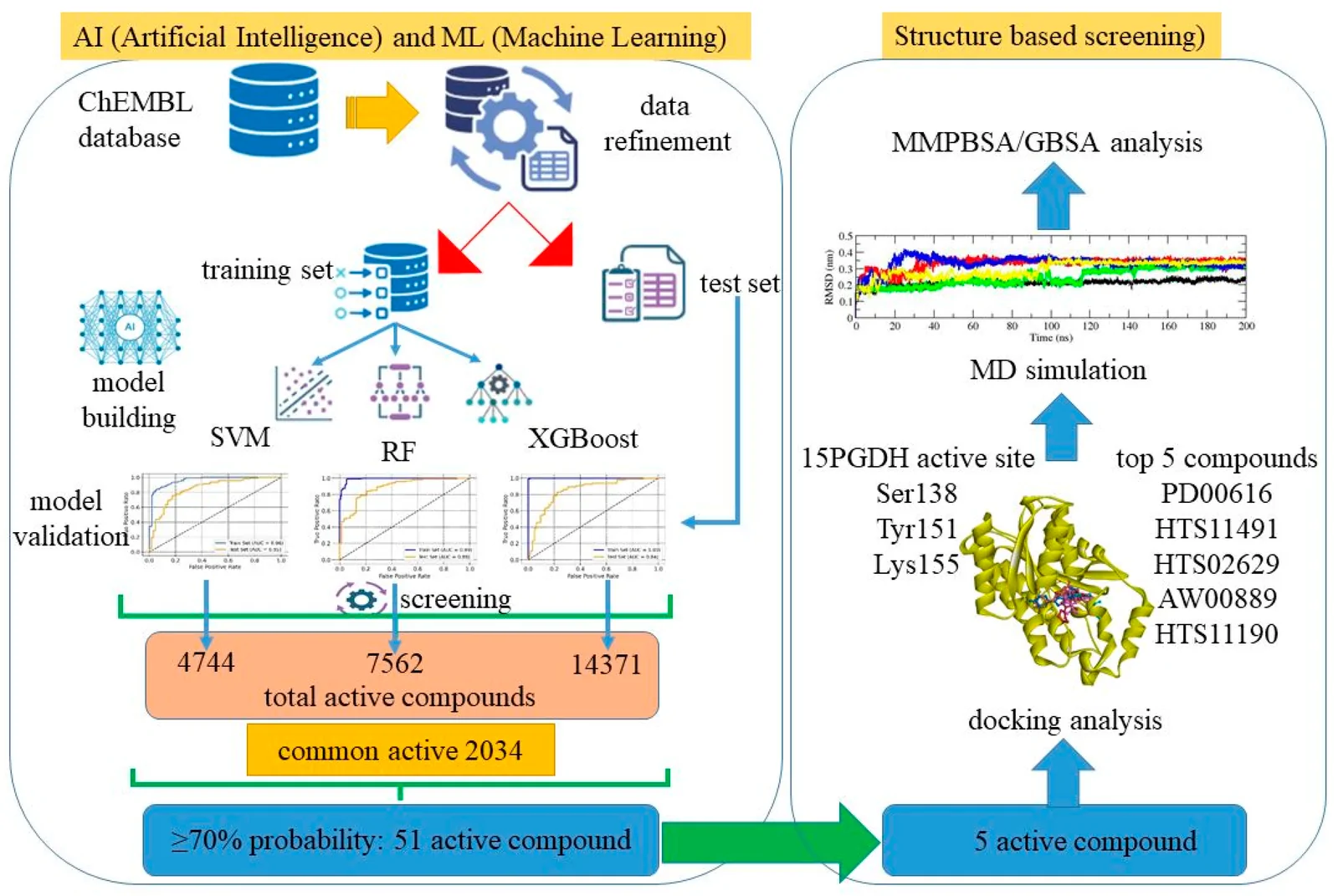
**Artificial intelligence, machine learning (ML), and structure-based virtual screening.** The known inhibitors of 15PGDH were retrieved from the ChEMBL database and curated to generate a refined dataset suitable for modeling. The processed dataset was subsequently divided into training and test sets for the development and validation of ML models: SVM, RF, and XGBoost. Compounds predicted with a probability score ≥ 70% were shortlisted for further structural and binding energy evaluation. These selected compounds were subjected to molecular docking, followed by MD simulations and MM-PBSA/GBSA binding free energy calculations to assess target–ligand interactions and binding stability.

The proposed study framework is shown in Figure 1. Compounds reported to exhibit activity against 15PGDH were retrieved from the ChEMBL database [14]. For activity assessment, the IC_50_ value reported for each compound was used as a measure of potency. The dataset was carefully curated to retain unique chemical structures, excluding any redundant entries, incomplete records, and chemically invalid molecules. For the development of a binary classification model, compounds with IC_50_ < 100 nM were labeled as active, whereas those with IC_50_ > 100 nM were labeled as inactive. The final dataset was split into training and test sets (70:30) for model development and validation.

### 2.2. Molecular Representations: Descriptors and Fingerprints Calculation

The SMILES (Simplified Molecular-Input Line-Entry System) strings of the training- and test-set inhibitors were used to identify descriptors. Molecular representations and feature extraction of these molecular descriptors were performed using PaDEL-Descriptor v2.0 [15], encompassing approximately 1875 one- and two-dimensional (1D/2D) molecular descriptors. Two molecular fingerprints, ECFP4 (1024 bits) and MACCS keys (166 bits) [16], were calculated using RDKit [17], and an Extended Connectivity Fingerprint-1024 (ECFP) [18] was generated using the Spyder package in conjunction with ML algorithms. Low-variance features were removed from the ChEMBL dataset to eliminate uninformative descriptors, reduce noise, improve model efficiency, and enhance predictive performance.

Fingerprints are binary or integer vectors that encode molecular substructures and are widely used in similarity searching, clustering, virtual screening, and ML-based drug discovery. Descriptors are numerical or categorical measures of molecular properties, including size, shape, hydrophobicity, and electronic distribution, which provide quantitative insight into how these features influence biological activity. ECFP captures atom-centered neighborhoods at multiple radii, reflecting both local and extended structural patterns. The 1024-bit version is highly effective in modern drug discovery. ECFP, a type of Morgan fingerprint with a radius of 2, decomposes inhibitors into atom-based substructures. As the radius expands, ECFP incorporates identifiers from previous and current iterations. These unique substructures are represented as 1024-bit vectors using one-hot encoding. Similarly, MACCS defines 166 substructures, which are also encoded using one-hot encoding.

### 2.3. Machine Learning Methods for Model Building

Before model development, the dataset was randomly split into a training set (70%) and a test set (30%). Supervised ML models were then developed using three algorithms: SVM [19], RF [20], and XGBoost [21]. For each algorithm, the optimal hyperparameter set was saved for model comparison and future predictions.

### 2.4. Support Vector Machine

SVM is a supervised ML algorithm that excels at binary classification tasks [19], such as predicting whether a molecule will inhibit a target. SVM uses kernel functions to transform high-dimensional chemical descriptor data for improved separability. Key hyperparameters include the regularization parameter C, which balances the decision margin and classification errors. A low C favors a broader margin, enhancing generalization, whereas a high C prioritizes correctly classifying the entire training set. Another critical parameter, gamma, controls the influence of individual training points. A small gamma yields a smoother decision boundary, whereas a large gamma produces a more complex boundary. Here, the optimized value of gamma was 0.0001, and the C value was 10.

### 2.5. Random Forest

RF [20] is a powerful ML technique that combines multiple decision trees to improve accuracy and generalization. RF leverages bootstrap sampling and random feature selection at each split, promoting diversity among trees and yielding a more robust model, particularly with high-dimensional data. To optimize performance, key hyperparameters such as the number of trees (n_estimators), maximum tree depth (max_depth), and the number of features considered at each split (max_features) must be carefully tuned to achieve the best results. The RF classifier was set as (n_estimators = 500, max_depth = 6, min_samples_split = 2, min_samples_leaf = 1, and max_features = ‘sqrt’).

### 2.6. XGBoost

XGBoost [21], an advanced ensemble learning algorithm, is ideal for classification tasks due to its capability of the algorithm to handle large datasets, manage missing values, and deliver strong performance on high-dimensional data. XGBoost was used to develop classification models, with model training and hyperparameter tuning aimed at optimizing prediction accuracy. Hyperparameter tuning was performed to improve performance, including adjustments to the learning rate, the number of estimators (n_estimators), and the maximum tree depth (max_depth). The XGBoost classifier was set with parameters (learning_rate = 0.004, max_depth = 12, n_estimators = 500) to develop the optimized model.

### 2.7. Model Evaluation

The predictive model was validated using various criteria, including statistical metrics such as accuracy, recall, precision, and F1 score, to assess model quality. Additionally, the performance was evaluated using the receiver operating characteristic (ROC) curve and the area under the curve (AUC) [22]. Predictions were generated for both the training and test sets to ensure robustness and generalizability. The AUC score provides a single measure of model performance, with values closer to 1 indicating better discrimination between classes. The following equations were used to analyze these parameters.

$$Recall=\frac{TP}{TP+FN}$$

$$Precision=\frac{TP}{TP+FP}$$

$$F1\;score=2\times \frac{Precision\times Recall}{Precision+Recall}$$

$$Accuracy=\frac{TP+TN}{TP+FP+FN+TN}$$

where TP: true positive, TN: true negative, FP: false positive, FN: false negative.

### 2.8. Applicability Domain Plot

To assess the reliability of the predictive models and delineate the chemical space, an applicability domain (AD) analysis was performed using a principal component analysis (PCA)-based approach [23,24]. The AD analysis was based on the molecular descriptors employed in model development. PCA was applied to the descriptor matrix to reduce dimensionality while retaining maximum variance. The first two principal components, which captured most of the variance, were then used to represent the compounds in a lower-dimensional space. A convex hull box was used to enclose the distribution of the training compounds within this PCA space.

### 2.9. Virtual Screening of Maybridge Library

The well-optimized selected models were used to identify potential hits against 15PGDH. Virtual screening was performed using the Maybridge library (http://www.maybridge.com (accessed on 10 February 2026)), which comprises ~51,000 molecules. Compounds predicted to be ‘active’ by all three models were retained. Hits were then selected in three steps: (1) identification of commonly predicted active compounds; (2) selection of compounds with a 70% probability score; and (3) inclusion of compounds within the AD plot of active compounds.

### 2.10. Molecular Docking Protocol

The crystal structure of 15PGDH (PDB ID: 2GDZ) [25], resolved at 1.65 Å, was obtained from the Protein Data Bank (www.rcsb.org/structure/2GDZ (accessed on 15 February 2026)) and prepared by removing water molecules, ligands, and heteroatoms using Discovery Studio (DS) 2021. The final selected compounds from the Maybridge library, identified using SVM, RF, and XGBoost, were screened against 15PGDH using PyRx 0.8 virtual screening software [26]. The PDB files of 15PGDH and the ligands were converted to PDBQT files for docking in PyRx. Additionally, multiple docking software, including EasyDockVina2.2 [27] and AutoDock4 [28], were used to further assess the accuracy and affinity of the top-ranked compounds.

### 2.11. Pharmacokinetic Analysis

Lipinski’s “Rule of Five” [29] was used to predict oral bioavailability and assess the therapeutic potential of the selected compounds. Most orally administered drugs have MWs < 500, logP values < 5, ≤5 H-bond-donating sites, and ≤10 H-bond-accepting sites. The selected compounds were also evaluated against additional drug-likeness rules, including the Ghose [30], Veber [31], Egan [32], and Muegge [33] criteria.

### 2.12. Molecular Dynamics Simulation

MD simulations of the best-ranked complexes were performed at 200 ns using GROMACS 2022 [34]. The 15PGDH topology was curated using the CHARMM force field [35]. The topologies of the selected compounds were generated using the SwissParam server [36]. The complex was solvated in a cubic box using the TIP3P water model [37]. The gmx genion module was used to neutralize the simulation box using Na^+^ and Cl^−^ ions. Bond lengths were constrained using the LINCS algorithm [38]. Equilibration phases were conducted for 100 ps under NVT (constant particle number, volume, and temperature) and NPT (constant particle number, pressure, and temperature). Finally, roots mean square deviations (RMSDs), root mean square fluctuations (RMSFs), radii of gyration (Rg), numbers of H-bonds, and solvent-accessible surface areas (SASAs) were analyzed.

### 2.13. MM-PBSA and MM-GBSA Analyses

The MM-PBSA/GBSA methods, integrated with GROMACS, were used to calculate binding energies [39]. These approaches are increasingly used to study biomolecular interactions and have recently been applied as scoring functions in computational drug design and discovery pipelines. The binding free energies between ligands and biological macromolecules can be determined using MM-PBSA [40] and MM-GBSA [41] methodologies. This study extracted stable molecular conformations from the equilibrium trajectories, enabling accurate measurement of the energies and stabilities of the target–ligand complexes based on the MD simulations. The binding free energy is calculated as follows:

ΔG_bind_ = G_com_ − (G_rec_ + G_lig_)

ΔE_MM_ = ΔE_internal_ + ΔE_electrostatic_ + ΔE_vdW_

ΔG_sol_ = ΔG_PB/GB_ + ΔG_SA_

ΔG_SA_ = γΔA + b

where ΔG_bind_ is the total free energy change between the bound state (G_com_) and unbound state (G_rec_ + G_lig_), and can be decomposed into: ΔE_MM_, gas-phase interaction energy, which contains electrostatic (ΔE_electrostatic_) and van der Waals (ΔE_vdW_) interactions; ΔG_sol_, the solvation energy, which includes the polar (ΔG_PB/GB_) and non-polar (ΔG_SA_) components.

## 3. Results

### 3.1. Molecular Descriptors and Chemical Space Analysis

To develop ML models for identifying novel inhibitors of 15PGDH, bioactivity data for compounds reported against 15PGDH were retrieved from the ChEMBL database and curated for model development. Compounds with reported biological activity, measured as IC_50_, were retrieved and curated. Then, the refined dataset was split into training and test sets to construct and train the SVM, RF, and XGBoost models. Training and test set instances were labeled as active if the IC_50_ value was ≤100 nM; all remaining compounds were labeled as inactive. The final training set comprised 364 compounds (211 active and 153 inactive), while the test set included 157 compounds (94 active and 63 inactive). To generate the models, compounds in the training set were processed using three molecular fingerprints (FPs): MACCS, ECFP, and 2D descriptors. Each fingerprint type was combined with each ML algorithm to build distinct classification models. Each model combination was then optimized to determine the best hyperparameter configuration for maximizing performance.

### 3.2. Machine Learning Model Development and Validation

The models were built using SVM, RF, and XGBoost, and the performance of each was evaluated (Table 1).

**Figure 2:**
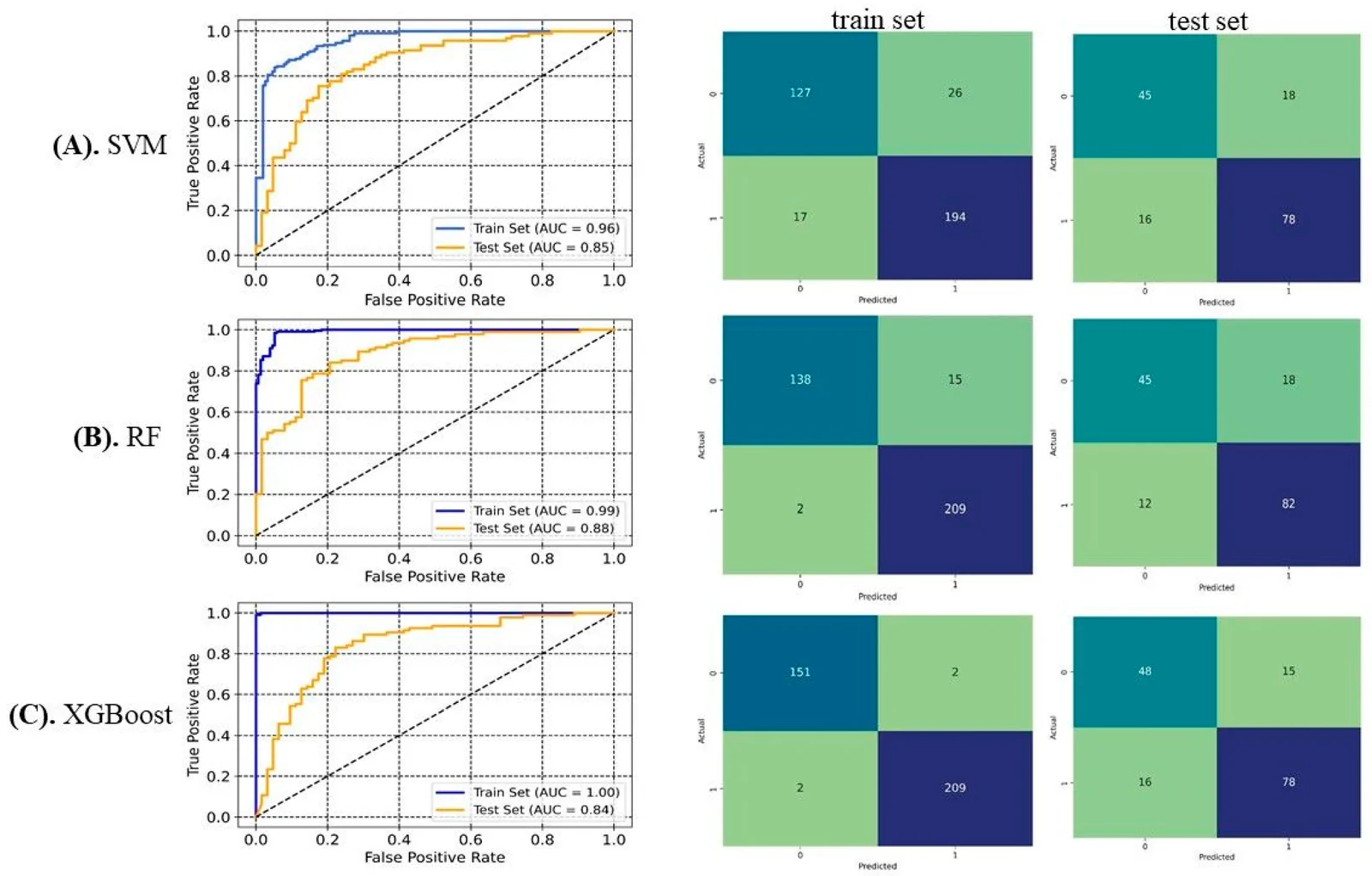
**Receiver operating characteristic (ROC) curves for the SVM, RF, and XGBoost models.** (**A**) SVM model with the area under the curve (AUC) value and corresponding confusion matrix. (**B**) RF model with the AUC and associated confusion matrix. (**C**) XGBoost model with the AUC and the confusion matrix, demonstrating the comparative predictive performance and classification efficiency of the three models.

Prediction performance was assessed using confusion matrices (overall accuracy, precision, recall, and F1 score) and ROC curves. SVM achieved accuracies of 0.88 and 0.78 on the training and test sets, respectively. On the test set, the recall, precision, and F1 scores for SVM were 0.92, 0.88, and 0.90, respectively (Figure 2A). RF achieved accuracies of 0.95 and 0.80 on the training and test sets, respectively. On the test set, the recall, precision, and F1 scores for RF were 0.99, 0.93, and 0.96, respectively (Figure 2B). XGBoost achieved accuracies of 0.98 and 0.80 on the training and test sets, respectively. On the test set, the recall, precision, and F1 scores for XGBoost were 0.83, 0.84, and 0.83, respectively (Figure 2C). The area under the receiver operating characteristic (AUROC) curve was used to further assess model robustness, demonstrating a strong balance between true- and false- positive rates and confirming the effectiveness of each method for the classification task. The AUCs for SVM (Figure 2A), RF (Figure 2B), and XGBoost (Figure 2C) were 0.96, 0.99, and 1.00, respectively, all of which are close to 1.

### 3.3. Screening of Maybridge Library

**Figure 3:**
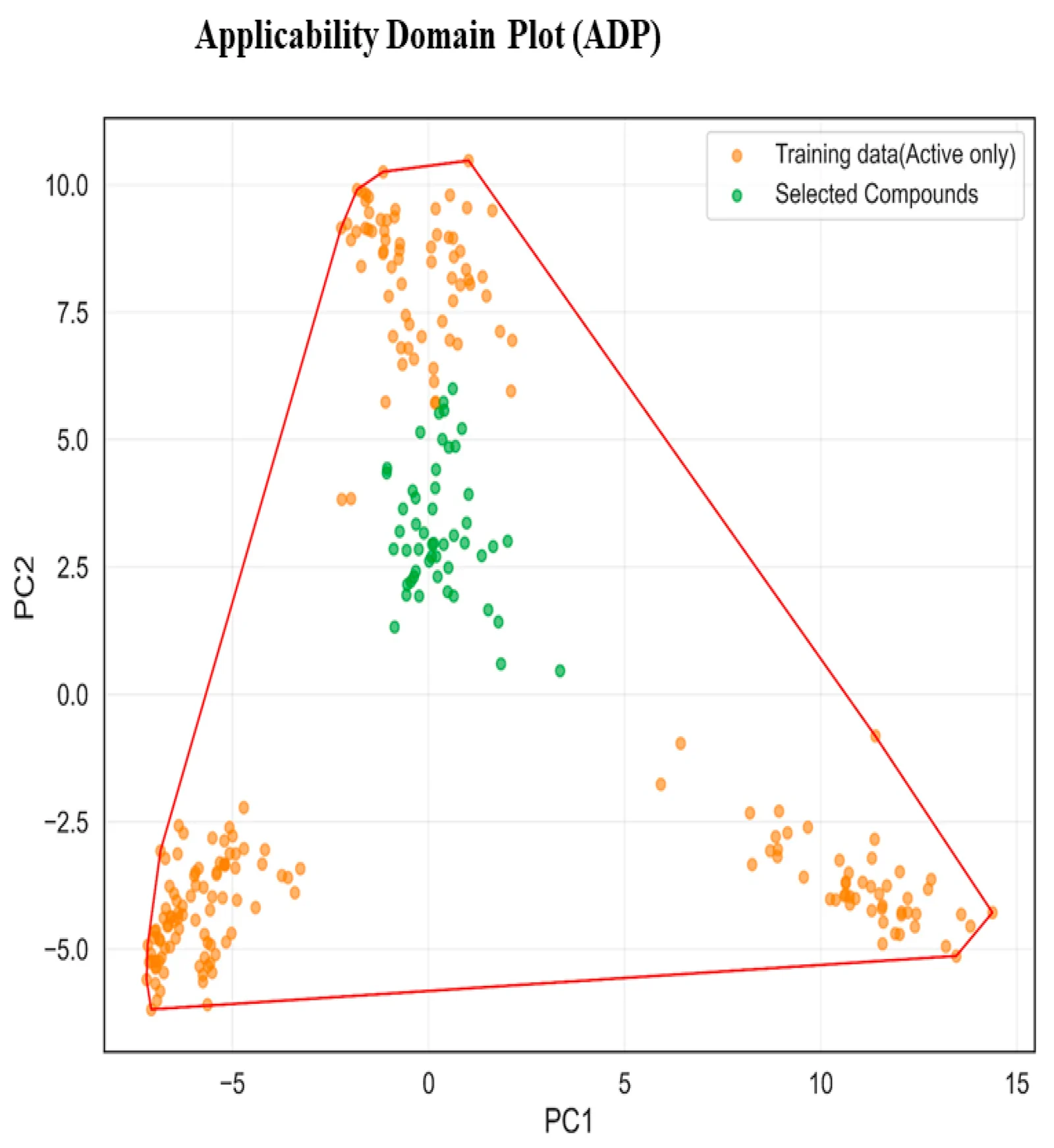
**Applicability domain (AD) plot.** A convex hull diagram of the chemical space for the training and top-selected compounds based on the first two principal components. The final selected compounds fall within the range of the training set.

The Maybridge library was used to screen the validated model. The SVM predicted 4744 compounds to be active, RF predicted 7562 compounds, and XGBoost predicted 14,371 compounds. Among these, 2034 active compounds were common across all three methods. Of these, 51 common compounds showed a ≥70% probability of being active across the ML-based models (Table 2). These 51 compounds were then assessed using an AD plot, which showed that the compounds fell within the AD defined by the active compounds in the training set (Figure 3). Finally, these compounds underwent further drug-likeness and ADMET analyses.

### 3.4. Drug-Likeness and ADMET Analysis

The top 51 compounds were subjected to drug-likeness and ADMET analyses using SwissADME [42]. After applying all these screening parameters, the top 5 compounds (Table 3), such as PD00616, HTS11491, HTS02629, AW00889, and HTS11190, with more than 70% probability of activity, were selected.

These compounds comply with all criteria for drug-like behavior (Table 4), including Lipinski’s “Rule of Five” (MWs < 500, logP < 5, ≤5 H-bond donors, and ≤10 H-bond acceptors) [29], as well as additional drug-likeness rules, such as Ghose [30], Veber [31], Egan [32], and Muegge [33]. Therefore, all five top compounds were selected for multi-score molecular docking (Table 3) and detailed MD simulations.

### 3.5. Docking Analysis

**Figure 4:**
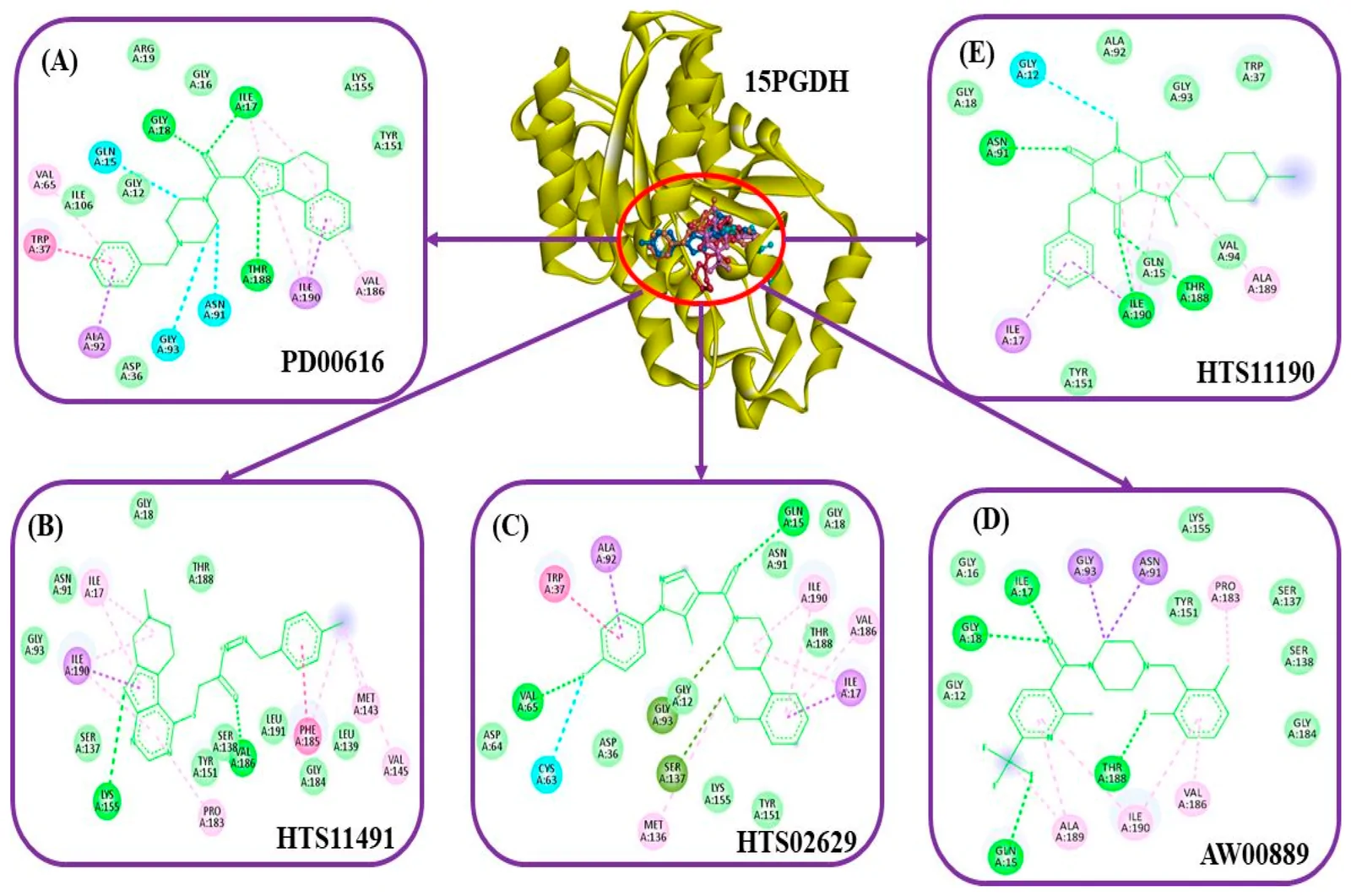
**Molecular docking interaction of the top five selected compounds against 15PGDH.** (**A**) PD00616 and 15PGDH, (**B**) HTS11491 and 15PGDH, (**C**) HTS02629 and 15PGDH, (**D**) AW00889 and 15PGDH, (**E**) HTS11190 and 15PGDH.

The PD00616 inhibitor was found to interact with several residues, including Gly12, Gln15, Gly16, Ile17, Gly18, Arg19, Asp36, Trp37, Val65, Asn91, Ala92, Gly93, Ile106, Tyr151, Lys155, Val186, Thr188, and Ile190 of 15PGDH, exhibiting a binding affinity of −11.0 kcal/mol. This affinity was further confirmed by other docking tools, such as AutoDock and EasyDockVina, which yielded high affinities of −8.94 and −10.5 kcal/mol, respectively (Table 3). In the PD00616–15PGDH complex, six H-bonds and nine hydrophobic interactions correctly position PD00616 within the 15PGDH active site (Table 5 and Table 6). In this interaction, PD00616 specifically engages key active-site residues, such as Tyr151 and Lys155, as well as surrounding residues (Figure 4A).

HTS11491 was found to interact with 15PGDH at residues Ile17, Gly18, Asn91, Gly93, Ser137, Ser138, Leu139, Met143, Val145, Tyr151, Lys155, Pro183, Gly184, Phe185, Val186, Thr188, and Ile190 (Figure 4B). HTS11491 binds specifically to the catalytic residues of 15PGDH, including Ser138, Tyr151, and Lys155. Additionally, two H-bonds and ten hydrophobic interactions contribute to the formation of a stable complex (Table 5 and Table 6). The binding affinity between HTS11491 and 15PGDH was −10.5 kcal/mol, which was further confirmed by AutoDock and EasyDockVina, which reported high affinities of −8.37 and −7.10 kcal/mol, respectively (Table 3).

HTS02629 was found to interact with several residues, including Gly12, Gln15, Ile17, Gly18, Asp36, Trp37, Cys63, Asp64, Val65, Asn91, Ala92, Gly93, Met136, Ser137, Tyr151, Lys155, Val186, Thr188, and Ile190 of 15PGDH, with a binding affinity of −10.5 kcal/mol. This affinity was further confirmed by other docking tools, such as AutoDock and EasyDockVina, which yielded high affinities of −8.16 and −9.90 kcal/mol, respectively (Table 3). In the HTS02629–15PGDH complex, four H-bonds (Table 5) and nine hydrophobic interactions (Table 6) correctly position HTS02629 within the 15PGDH active site. The types and distances of the H-bonds are reported in Table 5. In this interaction, HTS02629 specifically interacts with key active-site residues, such as Tyr151 and Lys155, as well as surrounding residues (Figure 4C).

AW00889 was found to interact with 15PGDH at amino acid residues Gly12, Gln15, Gly16, Ile17, Gly18, Asn91, Gly93, Ser137, Ser138, Tyr151, Lys155, Pro183, Gly184, Val186, Thr188, Ala189, and Ile190. AW00889 binds specifically to the catalytic residues of 15PGDH, including Ser138, Tyr151, and Lys155 (Figure 4D). Additionally, six H-bonds and six hydrophobic interactions contribute to the stability of the complex. The types and distances of the H-bonds are reported in Table 5, and hydrophobic interactions are listed in Table 6. The binding energy of AW00889 with 15PGDH was −9.40 kcal/mol and was further confirmed by AutoDock and EasyDockVina, which reported high affinities of −8.39 and −7.30 kcal/mol, respectively (Table 3).

HTS11190 was found to interact with 15PGDH at amino acid residues Gly12, Gln15, Ile17, Gly18, Trp37, Asn91, Ala92, Gly93, Val94, Tyr151, Thr188, Ala189, and Ile190 (Figure 4E). HTS11190 binds directly to the catalytic residue Tyr151 in 15PGDH. Additionally, four H-bonds and five hydrophobic interactions contribute to the formation of a stable complex. The types and distances of the H-bonds and hydrophobic interactions are shown in Table 5 and Table 6, respectively. The binding energy of HTS11190 with 15PGDH was −9.10 kcal/mol and was further confirmed by AutoDock and EasyDockVina, which showed high affinities of −8.62 and −9.40 kcal/mol, respectively (Table 3).

### 3.6. MD Simulation Analysis

MD simulations are used to analyze atomic movements, evaluate protein–ligand interaction stability, and confirm the rigidity of complexes under specific physiological conditions in computer-aided drug design [43]. The top-ranked complexes, such as PD00616–15PGDH, HTS11491–15PGDH, HTS02629–15PGDH, AW00889–15PGDH, and HTS11190–15PGDH, were subjected to MD simulations to assess their stability of the complexes over a 200 ns timescale, providing insights into their interactions and overall dynamics throughout the simulation period. The results for RMSD, RMSF, Rg, SASA, and H-bonds are summarized in Table 7.

#### 3.6.1. Root Mean Square Deviation Analysis

**Figure 5:**
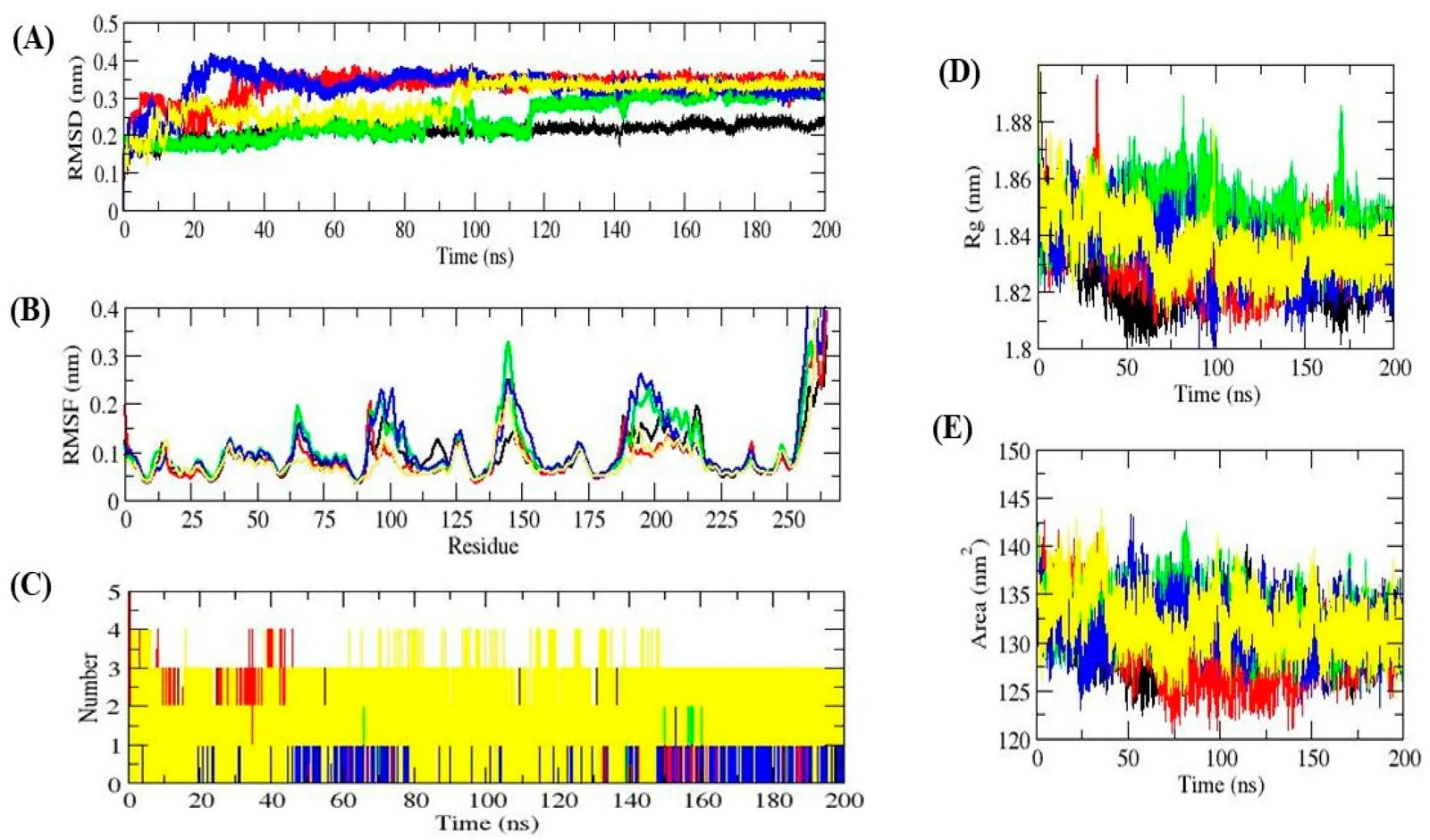
**MD simulation analysis of the top five selected complexes.** (**A**) Root mean square deviation (RMSD) analysis, (**B**) root mean square fluctuation (RMSF) analysis, (**C**) H-bond representations, (**D**) radii of gyration (Rg) analysis, and (**E**) solvent-accessible surface area (SASA) analysis of the complexes. These complexes were shown in different colors, such as PD00616–15PGDH (yellow color), HTS11491–15PGDH (green color), HTS02629–15PGDH (blue color), AW00889–15PGDH (black color), and HTS11190–15PGDH (red color).

RMSD analysis was performed to assess deviations in the target–ligand complex structures from the original conformations [44]. Figure 5A shows the RMSD profiles of the selected complexes, such as PD00616–15PGDH, HTS11491–15PGDH, HTS02629–15PGDH, AW00889–15PGDH, and HTS11190–15PGDH, as a function of simulation time (200 ns). All selected complexes remained stable throughout the simulation period, except for HTS02629–15PGDH, which became stable after 30 ns. The RMSD values of the selected complexes PD00616–15PGDH, HTS11491–15PGDH, HTS02629–15PGDH, AW00889–15PGDH, and HTS11190–15PGDH were 0.29 ± 0.04, 0.24 ± 0.05, 0.32 ± 0.04, 0.21 ± 0.01, and 0.32 ± 0.03 nm, respectively. Thus, the AW00889–15PGDH complex exhibited the lowest RMSD, although all complexes were considered stable.

#### 3.6.2. Root Mean Square Fluctuation Analysis

RMSF is used to identify residue flexibility upon ligand binding to the target over a simulation period of up to 200 ns [45]. Here, lower fluctuations were observed in the complexes at active-site residues and commonly interacting amino acids, including Ile17, Gly18, Asn91, Gly93, Tyr151, Thr188, and Ile190. Among these, Tyr151 exhibited the lowest fluctuation across all selected complexes (Figure 5B). The average RMSF values for the selected complexes of PD00616–15PGDH, HTS11491–15PGDH, HTS02629–15PGDH, AW00889–15PGDH, and HTS11190–15PGDH were 0.08 ± 0.07, 0.11 ± 0.08, 0.11 ± 0.07, 0.09 ± 0.04, and 0.08 ± 0.05 nm, respectively. Our analysis shows that the screened compounds PD00616, HTS11491, HTS02629, AW00889, and HTS11190 effectively interact with the active site of the target and contribute to complex stabilization. Overall, these top-screened compounds may serve as lead molecules for further drug design approaches. RMSF indicates protein residue mobility; higher values reflect greater atomic displacement, suggesting increased flexibility and instability in the receptor, whereas lower RMSF values suggest greater stability.

#### 3.6.3. H-Bond Analysis

H-bonds play an essential role in stabilizing protein–ligand complexes. We analyzed the number of H-bonds in these complexes over a 200 ns MD simulation period (Figure 5C). The results indicate that the PD00616–15PGDH complex has four, the HTS11491–15PGDH, HTS02629–15PGDH, and AW00889–15PGDH complexes each have three, and HTS11190–15PGDH has five H-bonds throughout the 200 ns simulation period.

#### 3.6.4. Radius of Gyration Analysis

The Rg is a key factor directly related to protein compactness, indicating how tightly a protein structure is folded. Figure 5D shows the Rg values for the PD00616–15PGDH, HTS11491–15PGDH, HTS02629–15PGDH, AW00889–15PGDH, and HTS11190–15PGDH complexes. Rg remains largely compact throughout the MD simulations of these protein–ligand complexes. The average Rg values for PD00616–15PGDH, HTS11491–15PGDH, HTS02629–15PGDH, AW00889–15PGDH, and HTS11190–15PGDH were 1.83 ± 0.01, 1.84 ± 0.009, 1.83 ± 0.01, 1.82 ± 0.01, and 1.83 ± 0.01 nm, respectively, over a 200 ns simulation time. A correctly folded protein maintains a stable Rg; thus, a lower Rg indicates greater compactness [46]. Here, all complexes displayed a consistently stable trajectory with only minor variation throughout the simulations.

#### 3.6.5. Solvent Accessible Surface Area Analysis

SASA is used to measure the ligand surface area exposed to solvent within a complex; a higher SASA value indicates a relatively wider surface area [47]. This value varies depending on the size and shape of the complex. The average SASA values for the selected complexes PD00616–15PGDH, HTS11491–15PGDH, HTS02629–15PGDH, AW00889–15PGDH, and HTS11190–15PGDH were 132.17 ± 2.74, 132.39 ± 2.37, 137.79 ± 2.44, 130.62 ± 2.46, and 129.70 ± 3.31 nm^2^, respectively (Figure 5E).

### 3.7. MM-PBSA/GBSA Analysis

The binding free energies of each system were calculated using MM-PBSA and MM-GBSA based on minimized structures. PB models were used to estimate polar desolvation in the MM-PBSA calculations. The total binding free energies for the selected complexes PD00616–15PGDH, HTS11491–15PGDH, HTS02629–15PGDH, AW00889–15PGDH, and HTS11190–15PGDH obtained by MM-PBSA were −33.71, −35.16, −24.90, −9.39, and −12.49 kcal/mol, respectively. The other energy components obtained by MM-PBSA, including ΔE_VDW_, ΔE_ELE_, ΔEgas, ΔG_PB_, ΔG_NPOLAR_, and ΔGsolv, are summarized in Table 8. The total binding free energies for the PD00616–15PGDH, HTS11491–15PGDH, HTS02629–15PGDH, AW00889–15PGDH, and HTS11190–15PGDH complexes obtained by MM-GBSA were −48.02, −40.01, −25.74, −22.46, and −12.58 kcal/mol, respectively. The corresponding energy components obtained by MM-GBSA are also shown in Table 8. These methods are important for the virtual screening and docking result improvement [48]. The total binding free energies obtained by the MM-PBSA and MM-GBSA methods are slightly different because these two methods use different implicit solvent models and electrostatic solvation energy calculations. MM-PBSA solves the Poisson–Boltzmann equation, whereas MM-GBSA uses the generalized Born approximation, which is computationally faster but often predicts more favorable (more negative) binding free energies. The slight difference in binding free energies between MM-PBSA and MM-GBSA arises from their distinct implicit solvent models. The Poisson–Boltzmann model numerically solves electrostatic interactions between solute and solvent, providing rigorous solvation energy estimates. In contrast, the generalized Born approximation analytically estimates electrostatic solvation, offering greater computational efficiency but generally predicting more favorable (more negative) binding free energies. Consequently, the absolute binding free energy values obtained from these methods are not expected to be identical as shown in Table 8. Here, a more negative total binding energy was estimated in MMGBSA as compared to MMPBSA, which supports the results mentioned by these two different methods, where total binding energy is greater in MMGBSA as compared to MMPBSA.

## 4. Discussion

SM is essential for mobility and body balance, but aging leads to sarcopenia, reducing muscle mass, strength, quality, and overall function. Age-related muscle degeneration is characterized by SM atrophy, leading to diminished function and structure [49,50]. PGE2 plays a pivotal role in maintaining MSC function and aiding muscle regeneration. However, with aging, the enzyme 15PGDH becomes more active, leading to reduced PGE2 levels. This reduction impairs muscle regeneration and exacerbates muscle loss, contributing to the decline in muscle health in older individuals [51]. Therefore, recent studies have investigated potential 15PGDH inhibitors to prevent excessive PGE2 degradation and alleviate 15PGDH-associated SM loss during aging, muscle atrophy, and sarcopenia. Here, an AI/ML-assisted structure-based drug design approach was employed to identify a new potential 15PGDH inhibitor. Recently, several studies applying AI/ML in drug designing have been published [52,53]. In this process, ML algorithms such as SVM, RF, and XGBoost were used to train models on data identified from the ChEMBL database. The AUROC curve values for the SVM, RF, and XGBoost models highlight the robustness of these models, indicating a strong balance between true positive and false positive rates and confirming the effectiveness of the models in the classification tasks. RF models are commonly used to assess the predictive performance of drug transporters, targets, and properties, and to measure the importance of feature descriptors [54,55]. The SVM model has been widely applied in cheminformatics for the discovery and design of novel drugs with excellent bioactivity [56]. XGBoost is also highly effective for a wide range of classification problems. The AUCs of the SVM, RF, and XGBoost models in this study were 0.96, 0.99, and 1.00, respectively, all approaching 1.

Once the models were built and optimized using the defined parameters, the Maybridge library (~51,000 compounds) was subjected to AI/ML-assisted virtual screening. A total of 4744 active compounds were predicted by SVM, 7562 by RF, and 14,371 by XGBoost, with 2034 compounds common across all methods. After applying a ≥70% probability threshold, 51 compounds were identified as active in all three ML-based screenings. The affinity of these 51 compounds for 15PGDH was assessed using the PyRx tool. The free energy of binding and the predicted activity probability obtained from SVM, RF, and XGBoost are reported in Table 2.

**Figure 6:**
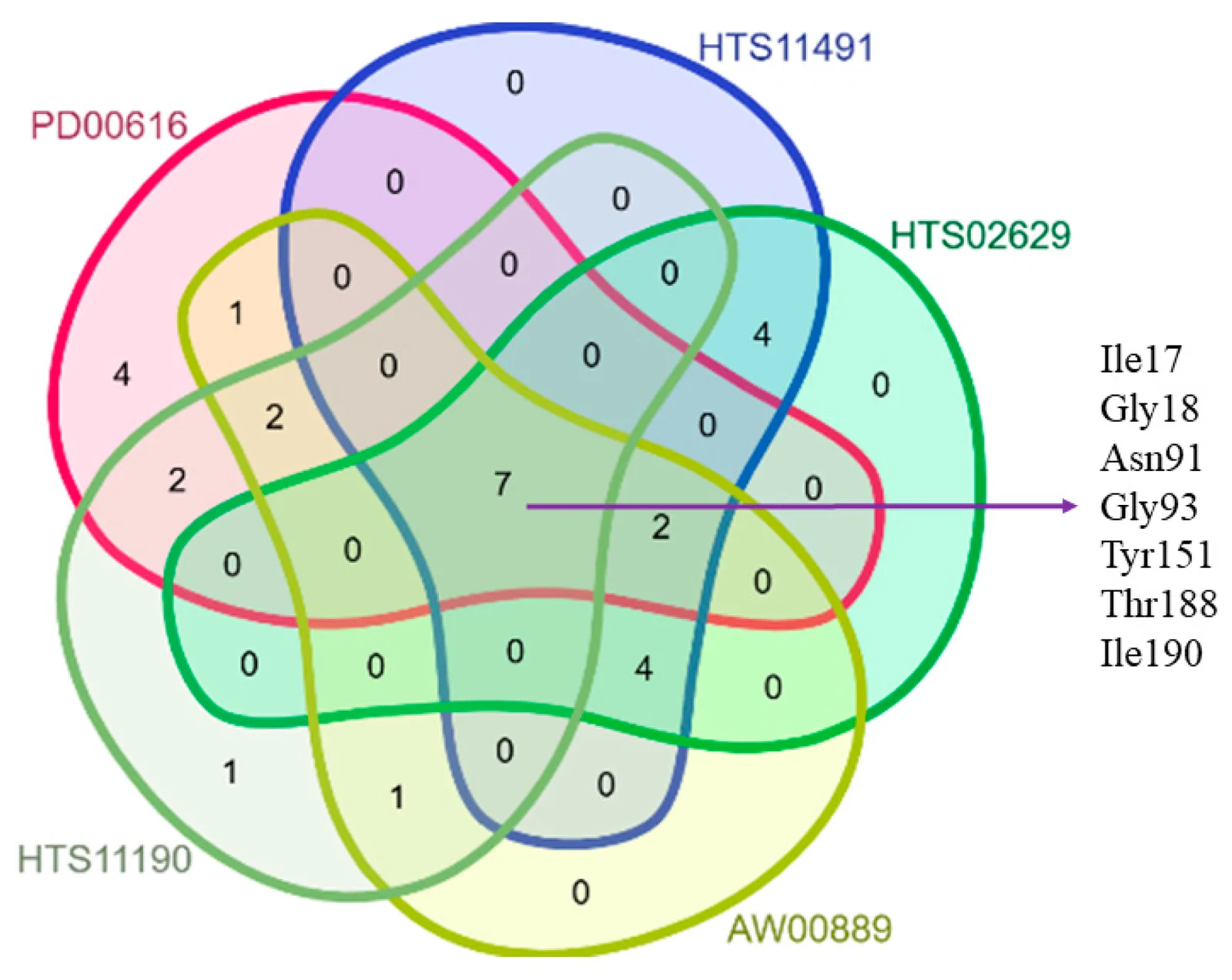
**Venn diagram.** The common amino acid residues among the top-selected compounds against 15PGDH. The catalytic residue Tyr151 was identified in all interactions, and its critical role in binding affinity was highlighted across all compounds.

These 51 compounds were subjected to drug-likeness analysis, from which five were deemed most suitable and largely met the criteria for drug-like behavior. The affinity of these top five compounds for 15PGDH was further assessed using multiple docking scores with AutoDock and EasyDockVina to validate the associated interaction affinity. Based on the free energy of binding, the top five compounds, namely, PD00616, HTS11491, HTS02629, AW00889, and HTS11190, exhibited the highest affinity for 15PGDH. Overall, in the selected complexes (PD00616–15PGDH, HTS11491–15PGDH, HTS02629–15PGDH, AW00889–15PGDH, and HTS11190–15PGDH), the amino acid residues Ile17, Gly18, Asn91, Gly93, Tyr151, Thr188, and Ile190 were commonly involved in the ligand–protein interactions (Figure 6). The binding free energies of these compounds, via PyRx, ranged from −9.10 to −11.0 kcal/mol, which is consistent with previously reported values [57], in which Sigmoidin B and Genistein showed binding affinities of −9.6 and −8.3 kcal/mol, respectively, for 15PGDH. In our previous study, molecular docking analysis demonstrated that the reference inhibitor, SW033291, which was used as the positive control due to its well-established high affinity for 15-PGDH had −8.00 kcal/mol of binding energy [9]. Additionally, the five highest-ranked compounds identified from the initial virtual screening were selected for multi-score molecular docking to improve the reliability of binding predictions. Employing multiple docking scoring functions enables a more comprehensive assessment of ligand–protein interactions, minimizes bias associated with a single scoring algorithm, and increases confidence in the predicted binding affinities and binding poses. This strategy facilitates the identification of the most promising computational hits against 15-PGDH.

Tyr151 and Lys155 were found in the interactions of PD00616 and HTS02629 with 15PGDH, whereas only Tyr151 was involved in the interaction of HTS11190 with 15PGDH, along with several other residues. Notably, HTS11491 and AW00889 interacted directly with all three catalytic triad residues (Ser138, Tyr151, and Lys155) in 15PGDH. Overall, Tyr151 is a key catalytic residue and, interestingly, is involved in all selected 15PGDH inhibitors.

Meanwhile, 15PGDH catalyzes the degradation of PGE2, which cannot bind to prostaglandin receptors. Inhibition of PGE2 signaling prevents MSC activation, thereby impairing SM regeneration and formation. The selected compounds may inhibit 15PGDH by binding to the associated catalytic residues, potentially restoring MSC function and enhancing myogenesis. This suggests that these compounds could stabilize MSC activity, promoting muscle regeneration. Thus, these compounds warrant further experimental evaluation as leads for 15PGDH inhibition and managing SM-related disorders.

Furthermore, based on binding free energy, amino acid residue interactions, and binding types, the top five complexes were selected for further validation via MD simulations at 200 ns. The aim was to assess the stability of these complexes within the 15PGDH active site. During the simulations, the complexes exhibited low RMSD and RMSF values, indicating stable binding and minimal structural deviations. The Rg analysis confirmed the compactness of the complexes, indicating stable folding within the active site. Consistently, SASA and H-bond analyses supported stability, showing stable binding interactions throughout the simulation. The two complexes HTS11491–15PGDH and AW00889–15PGDH exhibited lower RMSD values of 0.24 ± 0.05 nm and 0.21 ± 0.01 nm, respectively, while the others also fell within the acceptable range. Additionally, MM-PBSA/MM-GBSA have been successfully used to reproduce and rationalize experimental findings and refine virtual screening and docking results [48], with the results in good agreement regarding the stability of the complexes. The MM-PBSA and MM-GBSA provide a total binding free energies (ΔG_bind_) of −33.71 and −48.02 kcal/mol, respectively for the complex PD00616-15PGDH, while other complexes have similar energy profiles. The molecular mechanics (MM) components such as ΔE_VDW_ (−54.27 kcal/mol), ΔE_ELE_ (−24.28 kcal/mol), and ΔEgas (−78.55 kcal/mol) energy contributions were identical for both methods in each complex, indicating that the MM component was the same for both models. The observed difference in the total binding free energy arose exclusively from the solvation energy term. Specifically, the polar solvation energy calculated using the Poisson–Boltzmann model (49.61 kcal/mol) was substantially higher than that obtained by the Generalized Born approximation (36.98 kcal/mol), leading to a larger desolvation penalty in the MM-PBSA calculation for the complex PD00616-15PGDH. Consequently, MM-PBSA predicted a less favorable binding free energy than MM-GBSA. Such differences are expected because MM-PBSA numerically solves the Poisson–Boltzmann equation, whereas MM-GBSA employs an analytical approximation. Here, the polar solvation energy is the major contributor, such that MM-PBSA (ΔG_PB_) was 49.61 kcal/mol and MM-GBSA (ΔG_GB_) was 36.98 kcal/mol in the same complex. It is reported that PB predicts a stronger desolvation penalty, giving a less negative ΔG_bind_, while GB predicts a smaller desolvation penalty, which gives a more negative ΔG_bind_. The Poisson–Boltzmann model numerically solves electrostatic interactions between solute and solvent, providing rigorous solvation energy estimates, whereas the generalized born approximation analytically estimates electrostatic solvation, offering greater computational efficiency but typically predicting more favorable binding free energies [48,58]. Importantly, both methods consistently predict favorable binding of PD00616 to 15PGDH, supporting the stability of the protein–ligand complex. These findings suggest that the selected compounds maintain stable interactions within the 15PGDH active site, supporting the potential use of these compounds as effective 15PGDH inhibitors. Notably, 15PGDH inhibition has shown promise in enhancing skin wound healing [59], tissue regeneration [10], and mitigating conditions such as muscular dystrophy [60] and other diseases.

## 5. Limitations

Although this study provides valuable insights into computational hits targeting 15-PGDH, several limitations should be considered when interpreting the findings. First, all conclusions are based on computational approaches, including AI/ML-assisted screening, including virtual screening, molecular docking, MD simulations, and ADME predictions. While these methods offer valuable information regarding binding affinity, stability, and pharmacokinetic properties, they cannot fully replicate the complexity of biological systems. Therefore, experimental validation through enzymatic inhibition assays or cell-based studies is necessary to confirm the predicted binding interactions, inhibitory activity, efficacy, and safety of the identified compounds before considering their therapeutic potential.

## 6. Future Work

The findings of this study provide a computational framework for the identification of 15-PGDH computational hits; however, comprehensive experimental validation is required before clinical translation. Future investigations should begin with in vitro enzymatic inhibition assays to determine the IC_50_ of the identified compounds against 15-PGDH. Subsequently, their biological activity should be evaluated in relevant SM cell models by assessing target engagement, myogenic differentiation, cell viability, and potential cytotoxicity. Compounds demonstrating favorable activity should undergo pharmacokinetic, metabolic stability, and selectivity profiling, followed by efficacy evaluation in established animal models of SM atrophy or age-related sarcopenia. Collectively, these studies will validate computational predictions and facilitate the development of safe and effective 15-PGDH-targeted therapeutics for improving SM regeneration and function.

## 7. Conclusions

ML methods, including SVM, RF, and XGBoost, were used to develop models for identifying potential 15PGDH inhibitors, thereby improving drug discovery efficiency and accelerating targeted therapy development. These computational models were further integrated with molecular docking and MD simulations to validate binding affinity, complex stability, and interaction profiles. This comprehensive in silico framework provides a reliable and efficient strategy for the discovery and optimization of novel 15PGDH inhibitors. Through these approaches, we screened and identified five promising compounds, namely PD00616, HTS11491, HTS02629, AW00889, and HTS11190, as potent inhibitors of 15PGDH. These findings offer valuable insights for the development of novel therapeutics targeting 15PGDH to combat muscle degeneration and related pathologies, including aging and sarcopenia.

## Figures and Tables

**Table 1:** The performance of the SVM, RF, and XGBoost models in terms of their accuracy, recall, precision, F1 scores, and AUC of the developed classification models.

Process	Data Set	Accuracy	Recall	Precision	F1 Score	AUC
SVM	Training Set	0.88	0.92	0.88	0.90	0.96
	Test Set	0.78	0.83	0.78	0.73	0.85
RF	Training Set	0.95	0.99	0.93	0.96	0.99
	Test Set	0.80	0.87	0.82	0.84	0.88
XGBoost	Training Set	0.98	0.99	0.99	0.99	1.00
	Test Set	0.80	0.83	0.84	0.83	0.84

**Table 2:** The list of compounds having more than 70% of probability in SVM, RF, and XGBoost ML based screening.

Compound ID	SVM	RF	XGBoost	PyRx
SPB05208	0.95	0.74	0.81	−7.8
SPB07226	0.94	0.70	0.85	−10.8
SEW06515	0.93	0.77	0.74	−12.4
AW01067	0.91	0.70	0.79	−11.0
SEW06281	0.91	0.72	0.70	−10.4
HTS10845	0.91	0.70	0.70	−8.7
KM05106	0.90	0.74	0.82	−8.9
SP01058	0.89	0.70	0.71	−10.7
KM05095	0.89	0.71	0.80	−8.7
SEW06283	0.88	0.70	0.72	−11.1
HTS11190	0.88	0.72	0.72	−9.1
RJF01813	0.88	0.70	0.80	−7.5
SPB07228	0.88	0.71	0.82	−10.2
AW00483	0.88	0.74	0.78	−7.8
HTS01814	0.86	0.71	0.89	−9.2
HTS03786	0.86	0.74	0.90	−10.7
HTS00674	0.86	0.72	0.88	−10.5
HTS03276	0.85	0.76	0.85	−7.9
AW00889	0.85	0.73	0.86	−9.4
RH01577	0.84	0.76	0.85	−9.1
SPB07057	0.84	0.71	0.79	−7.1
HTS02629	0.83	0.75	0.90	−10.5
AW01005	0.82	0.76	0.84	−9.7
SEW06514	0.82	0.73	0.83	−11.6
HTS01157	0.82	0.71	0.75	−7.7
RH01519	0.82	0.71	0.79	−7.6
KM03869	0.81	0.70	0.76	−8.6
KM08113	0.80	0.70	0.79	−8.6
KM11066	0.79	0.72	0.87	−9.3
AW01085	0.78	0.70	0.76	−8.1
SP01050	0.78	0.71	0.90	−9.3
SP00413	0.78	0.74	0.90	−7.7
KM10702	0.77	0.70	0.83	−7.7
HTS11491	0.77	0.72	0.89	−10.5
RJF01612	0.77	0.70	0.72	−7.3
RH01613	0.77	0.70	0.87	−10.8
JFD01697	0.76	0.71	0.70	−8.6
CD02150	0.76	0.70	0.87	−9.7
SEW06516	0.76	0.71	0.71	−11.11
KM05188	0.76	0.70	0.74	−7.0
JP00647	0.75	0.72	0.72	−7.2
KM05197	0.74	0.73	0.73	−7.1
BTB02229	0.73	0.70	0.80	−7.1
KM04252	0.73	0.73	0.86	−8.3
KM11112	0.73	0.70	0.80	−8.8
HTS03278	0.73	0.70	0.79	−11.7
KM05198	0.71	0.72	0.79	−7.4
PD00616	0.71	0.71	0.77	−11.0
KM08831	0.71	0.70	0.87	−8.9
KM09459	0.71	0.74	0.87	−7.9
KM09481	0.70	0.70	0.84	−6.4

**Table 3:** List of top five compounds having more than 70% probability through ML-models and their multi score docking (in kcal/mol) analysis.

Compound ID	SVM	RF	XGBoost	EasyDockVina2	PyRx	Autodock
HTS11190	0.88	0.72	0.72	−9.4	−9.1	−8.62
AW00889	0.85	0.73	0.86	−7.3	−9.4	−8.39
HTS02629	0.83	0.75	0.90	−9.9	−10.5	−8.16
HTS11491	0.77	0.72	0.89	−7.1	−10.5	−8.37
PD00616	0.71	0.71	0.77	−10.5	−11.0	−8.94

**Table 4:** The molecular weight and drug-likeness rules of the top five selected compounds.

Compound	Molecular Weight (g/mol)	Formula	Drug-Likeness Rules				
			Lipinski	Veber	Egan	Muegge	Ghose
PD00616	388.53	C_24_H_24_N_2_OS	Yes	Yes	Yes	Yes	Yes
HTS11491	430.97	C_20_H_19_ClN_4_OS_2_	Yes	Yes	Yes	No	Yes
HTS02629	393.45	C_23_H_24_FN_3_O_2_	Yes	Yes	Yes	Yes	Yes
AW00889	415.81	C_19_H_18_ClF_4_N_3_O	Yes	Yes	Yes	Yes	Yes
HTS11190	446.34	C_20_H_24_BrN_5_O_2_	Yes	Yes	Yes	Yes	Yes

**Table 5:** H-bond analysis of the top five selected compounds with 15PGDH.

Compounds	H-Bond	Distance	Type
PD00616	ILE17:HN-UNL1:O1	2.42	Conventional H-Bond
	GLY18:HN-UNL1:O1	1.86	
	THR188:HG1-UNL1:S1	2.68	
	UNL1:C14-ASN91:O	3.71	Carbon H-Bond
	UNL1:C15-GLY93:O	3.20	
	UNL1:C24-GLN15:O	3.54	
HTS11491	LYS155:HZ1-UNL1:S2	2.88	Conventional H-Bond
	VAL186:HN-UNL1:O1	1.84	
HTS02629	GLN15:HE22-UNL1:O1	2.77	Conventional H-Bond
	VAL65:HN-UNL1:F1	2.28	
	UNL1:C16-GLY93:O	3.30	Carbon H-Bond
	UNL1:C23-SER137:O	3.53	
AW00889	GLN15:HE21-UNL1:F1	2.77	Conventional H-Bond
	ILE17:HN-UNL1:O1	2.80	
	GLY18:HN-UNL1:O1	2.76	
	THR188:HG1-UNL1:F4	2.36	
	UNL1:C9-ASN91:O	3.22	Carbon H-Bond
	UNL1:C9-GLY93:O	3.53	
HTS11190	ASN91:HD21-UNL1:O1	2.14	Conventional H-Bond
	THR188:HG1-UNL1:O2	2.78	
	ILE190:HN-UNL1:O2	2.33	
	UNL1:C20-GLY12:O	3.66	Carbon H-Bond

**Table 6:** Hydrophobic interaction analysis of the top five selected compounds with 15PGDH.

Compounds	Hydrophobic Interaction	Type
PD00616	ALA92:CB-UNL1	Pi-Sigma
	ILE190:CG2-UNL1	
	TRP37-UNL1	Pi-Pi Stacked
	ILE17-UNL1	Alkyl
	ILE190-UNL1	
	UNL1-ILE17	Pi-Alkyl
	UNL1-ILE190	
	UNL1-VAL186	
	UNL1-VAL65	
HTS11491	ILE190:CD1-UNL1	Pi-Sigma
	PHE185-UNL1	Pi-Pi Stacked
	ILE17-UNL1	Alkyl
	ILE190-UNL1	
	UNL1:CL1-MET143	
	UNL1:CL1-VAL145	
	PHE185-UNL1:CL1	Pi-Alkyl
	UNL1-PRO183	
	UNL1-ILE190	
	UNL1-ILE17	
HTS02629	ILE17:CD1-UNL1	Pi-Sigma
	ALA92:CB-UNL1	
	TRP37-UNL1	Pi-Pi Stacked
	TRP37-UNL1	
	ILE17-UNL1	Alkyl
	ILE190-UNL1	
	UNL1:C23-MET136	
	UNL1-VAL186	Pi-Alkyl
	UNL1-ILE190	
AW00889	ALA189-UNL1:C8	Alkyl
	UNL1:CL1-PRO183	
	UNL1-ALA189	Pi-Alkyl
	UNL1-ILE190	
	UNL1-VAL186	
	UNL1-ILE190	
HTS11190	ILE17:CD1-UNL1	Pi-Sigma
	ILE190:CD1-UNL1	
	UNL1-ILE190	Pi-Alkyl
	UNL1-ALA189	
	UNL1-ILE190	

**Table 7:** MD simulation analysis of the top five selected complexes.

Complex	RMSD	Rg	SASA	H-Bond
AW00889-15PGDH	0.21 ± 0.01	1.82 ± 0.01	130.62 ± 2.46	3
HTS11190-15PGDH	0.32 ± 0.03	1.83 ± 0.01	129.7 ± 3.31	5
HTS11491-15PGDH	0.24 ± 0.05	1.84 ± 0.009	132.39 ± 2.37	3
HTS02629-15PGDH	0.32 ± 0.04	1.83 ± 0.01	137.79 ± 2.44	3
PD00616-15PGDH	0.29 ± 0.04	1.83 ± 0.01	132.17 ± 2.74	4

**Table 8:** The binding free energy of the top five selected complexes through MM-PBSA/GBSA analysis in kcal/mol.

**MM-PBSA**							
**Complex**	**ΔE_VDW_**	**ΔE_ELE_**	**ΔE_gas_**	**ΔG_PB_**	**ΔG_NPOLAR_**	**ΔG_solv_**	**ΔG_bind_**
AW00889-15PGDH	−38.06	−12.78	−50.84	46.01	−4.55	41.45	−9.39
HTS11190-15PGDH	−20.58	−3.32	−23.9	14.04	−2.62	11.42	−12.49
HTS11491-15PGDH	−45.24	−169.99	−215.24	184.83	−4.75	180.08	−35.16
HTS02629-15PGDH	−40.46	−9.46	−49.92	29.86	−4.85	25.02	−24.9
PD00616-15PGDH	−54.27	−24.28	−78.55	49.61	−4.77	44.84	−33.71
**MM-GBSA**							
**Complex**	**ΔE_VDW_**	**ΔE_ELE_**	**ΔE_gas_**	**ΔG_GB_**	**ΔG_Esurf_**	**ΔG_solv_**	**ΔG_bind_**
AW00889-15PGDH	−38.06	−12.78	−50.84	33.81	−5.44	28.37	−22.46
HTS11190-15PGDH	−20.58	−3.32	−23.9	14.14	−2.81	11.32	−12.58
HTS11491-15PGDH	−45.24	−169.99	−215.24	181.47	−6.24	175.22	−40.01
HTS02629-15PGDH	−40.46	−9.46	−49.92	29.48	−5.3	24.18	−25.74
PD00616-15PGDH	−54.27	−24.28	−78.55	36.98	−6.46	30.53	−48.02

## Data Availability

All the data are included within the manuscript.

## Abbreviations

SM	Skeletal muscle
MSCs	Muscle satellite (stem) cells
PGE2	Prostaglandin E2
15PGDH	15-hydroxyprostaglandin dehydrogenase
ML	Machine learning
AUC	Area under the curve
